# Characterization of murine mammary stem/progenitor cells in a D-galactose-induced aging model

**DOI:** 10.18632/aging.202870

**Published:** 2021-04-20

**Authors:** Hui Gao, Yahui Liu, Min Zheng, Fan Zhao, Hongzhu Wang, Jiajian Yu, Hao Jiang, Danhan Wang, Qiaoxiang Dong

**Affiliations:** 1 The Second Affiliated Hospital and Yuying Children's Hospital, Wenzhou Medical University, Wenzhou 325035, PR China; 2 Institute of Environmental Safety and Human Health, Wenzhou Medical University, Wenzhou 325035, PR China; 3 Key Laboratory of Fertility Preservation and Maintenance of Ministry of Education, Ningxia Medical University, Yinchuan 750004, PR China

**Keywords:** aging, mammary stem/progenitor cells, D-galactose, breast cancer

## Abstract

Aging plays an important role in many diseases, including breast cancer. Aged mammary stem/progenitor cells are perceived to be the cells of origin in breast tumorigenesis; however, the extensive use of mice who have aged naturally for research is hampered by cost, time, disease complications, and high mortality. In this study, we characterized murine mammary stem/progenitor cells in a D-galactose-induced accelerated aging model and compared them with findings from our earlier study on mice from natural aging. Our results showed that mammary glands in the D-galactose-induced aging model mimic natural aging in terms of pathological changes, epithelial cell composition, and mammary stem/progenitor cell function. These changes are accompanied by elevated inflammatory responses both systemically in the blood and locally in the mammary glands, which is similar in mice who age naturally. Our study for the first time evaluated the mammary glands and mammary stem/progenitor function in a D-galactose-induced aging model in rodents, and our findings suggest that D-galactose treatment can be used as a surrogate to study the role aged stem/progenitor cells play in breast tumorigenesis.

## INTRODUCTION

Breast cancer is one of the leading causes of death in female cancer patients with nearly 630,000 deaths and 2.1 million new cases diagnosed worldwide each year [1]. Of all breast cancer risk factors, aging is the most prominent, as more than 80% of women are diagnosed at > 50 years old, and the incidence of invasive breast cancer increases exponentially with age [2, 3]. Aging also plays an important role in women with a family history of breast cancer [4]. More recently, higher p16^INK4a^ mRNA expression in T cells was found to be a risk factor for breast cancer [5]. As p16^INK4a^ is a marker of cellular senescence, which is a hallmark of aging, this finding further supports the importance of biological aging in the etiology of breast cancer development. Despite this, few studies have incorporated aging as an important factor in mammary tumorigenesis.

Mammary stem/progenitor cells are perceived to be the cells of origin in breast cancer given their relatively long-lived, self-renewal and multi-lineage differentiation properties, which are shared by cancer initiating cells [6–10]. Gene expression analysis revealed correlations between different stem/progenitor cells with different breast cancer subtypes [11]. Many studies have shown that inhibitors that depleted mammary stem/progenitors also delayed aggressive breast cancer tumorigenesis [12]. Coupled with the aging risk factor, mammary stem/progenitor cells are likely to be vulnerable targets for tumorigenesis. In fact, we have demonstrated that aged mammary stem cells have increased neoplastic transformation potential [13]. The use of aged stem/progenitor cells may therefore help us better understand breast cancer initiation, progress, and metastasis.

Naturally aged mice are the ideal model for studying aged mammary stem/progenitor cells; however, extensive application of this model is not only expensive and time-consuming, but also subject to disease complications and high mortality [14, 15]. Rodents chronically injected with D-galactose for a period of 6–10 weeks showed progressive functional decline in multiple organs and have been used as an accelerated aging model [14, 16]. *In vivo*, D-galactose treatment successfully mimicked the natural aging process in terms of increased oxidative stress, proinflammatory cytokines, and persistent chronic inflammation in multiple organ systems such as the brain, heart, lungs, liver, kidney, reproductive system, auditory system, skin, bones, skeletal muscle, and immune system [17]. *In vitro*, D-galactose-treated cells manifested aging characteristics such as increased oxidative stress, inflammation, senescence-associated beta-galactosidase (SA-β-gal) staining, up-regulated p16, p53, and p21, and down-regulated nuclear factor erythroid 2-related factor 2 (NRF2) and heme oxygenase-1 (HO-1) [17]. In contrast to natural aging, the D-galactose-induced aging model also has the advantages of convenience, the least side effects, and a high survival rate throughout the experimental period [18, 19]. Since its introduction in 1985, D-galactose has been widely used in various aging studies [17, 20]. However, its use in studying how aging might affect mammary tumorigenesis has not been explored.

In this study, we characterized murine mammary stem/progenitor cells in a D-galactose-induced aging model and compared them with findings from our earlier study on mice subject to natural aging.

## RESULTS

### D-galactose induces elevated inflammatory signature

**Figure 1:**
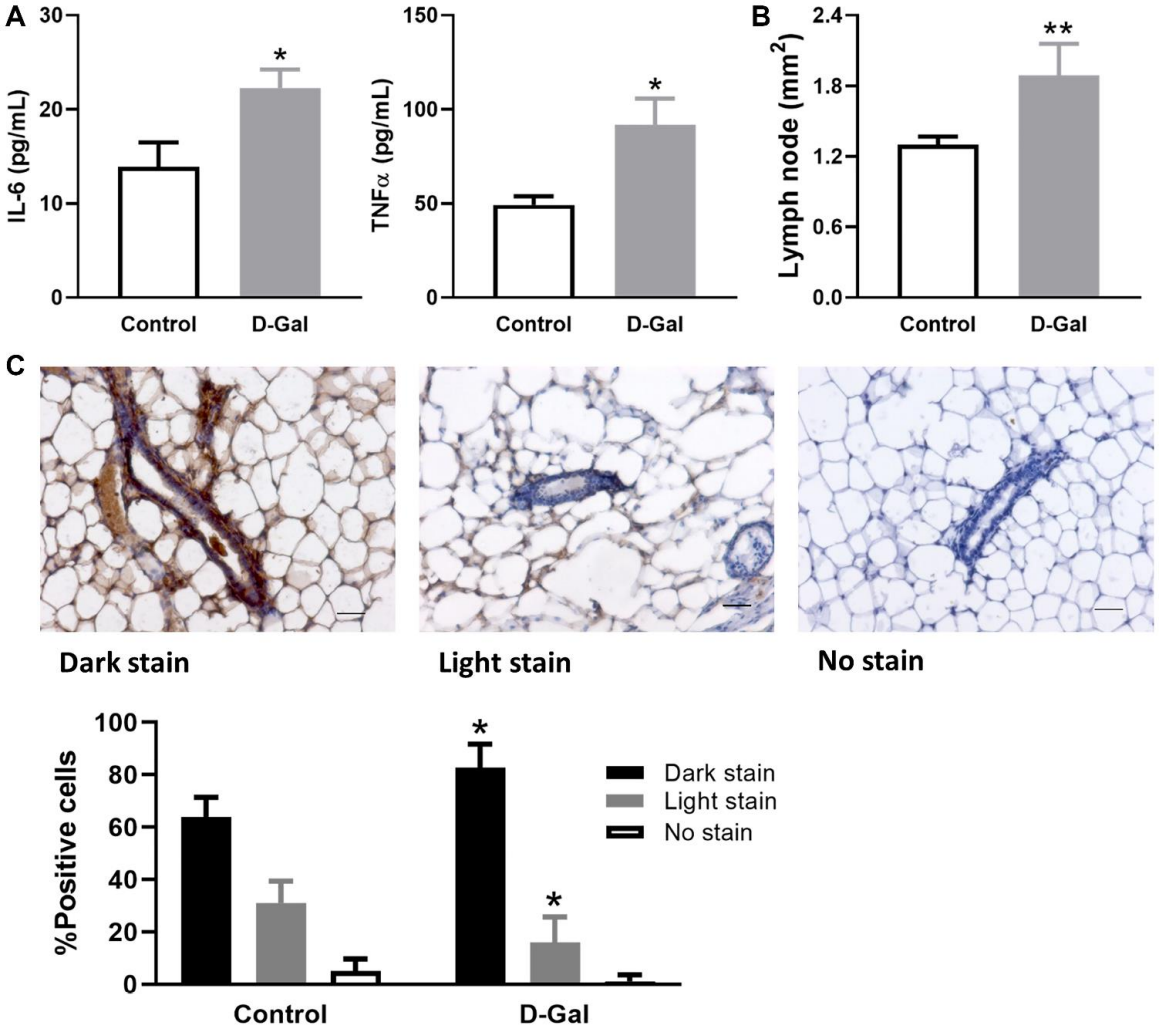
**D-galactose induces elevated inflammatory signature.** (**A**) Levels of inflammatory cytokine IL-6 and TNFα in serum samples collected from control mice and those treated with D-galactose (*n* = 3); (**B**) The area of lymph nodes in the mammary glands from control and D-galactose-treated mice (*n* = 5); (**C**) Quantification of p-STAT5 staining in mammary glands from control and D-galactose-treated mice (*n* = 4). Scale bars, 50 μm. Asterisks, significant difference between control and D-galactose (^*^P < .05, ^**^P < .01).

The mice treated with D-galactose exhibited significantly higher plasma levels of inflammatory cytokine IL-6 and TNFα than the control group (Figure 1A). Specifically, the levels of IL-6 and TNFα were increased 1.6- and 1.9-fold, respectively. Mammary glands from D-galactose-treated mice had enlarged lymph nodes, which on average increased approximately 1.45-fold when measured by area (Figure 1B). In addition, p-STAT5, a downstream regulator of IL-6 and TNFα, was detected at a higher frequency of dark staining in mammary glands from D-galactose-treated mice (83%) than those from control mice (64%) (Figure 1C). The D-galactose group also demonstrated a significantly lower level of p-STAT5 light staining than the control (16% vs. 31%) (Figure 1C).

### D-galactose decreases branching point and increases hyperplasia in mammary glands

**Figure 2:**
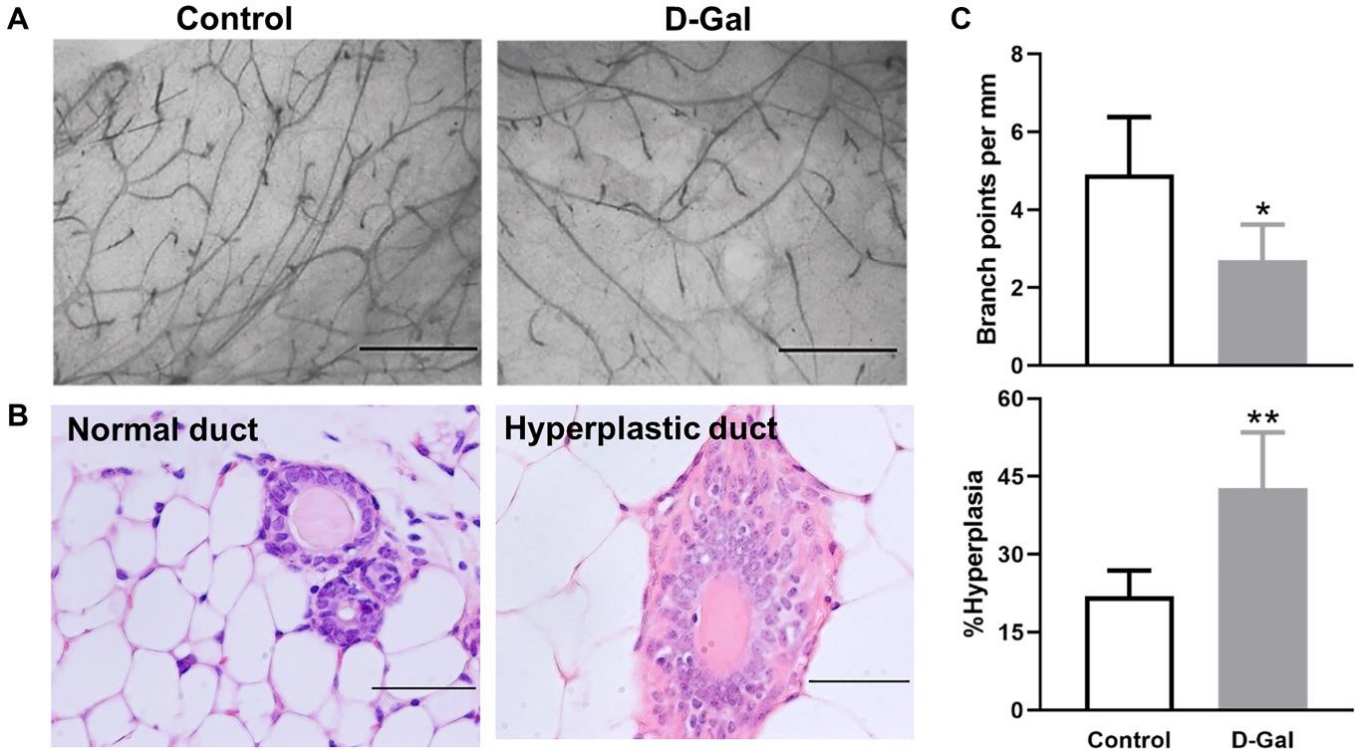
**D-galactose affects mammary gland morphology and pathology.** (**A**) Representative examples of whole mount carmine alum staining of mammary glands from control and D-galactose-treated mice (scale bars, 1 mm); (**B**) H&E histological images showing normal and hyperplastic ducts from control and D-galactose-treated mice (scale bars, 100 μm); (**C**) Quantification of branch points per millimeter (mm) duct (upper panel) and percent of hyperplastic ducts (lower panel) in control and D-galactose-treated mice (*n* = 5). Asterisks, significant difference between control and D-galactose (^*^P < .05, ^**^P < .01).

Mice treated with D-galactose showed altered mammary glands that were characterized by decreased branching points and higher frequency of hyperplastic mammary ducts (Figure 2). The branching points per millimeter (mm) mammary duct were decreased, with 4.9 ± 1.5 in control mice and 2.7 ± 0.9 in D-galactose-treated mice (*P* < 0.05) (Figure 2A, 2C). The frequency of hyperplastic duct increased from 21.9 ± 4.9% in control mice to 42.8 ± 10.1% in D-galactose-treated mice (*P* < .01) (Figure 2B, 2C).

### D-galactose alters mammary basal/luminal cell pools

**Figure 3:**
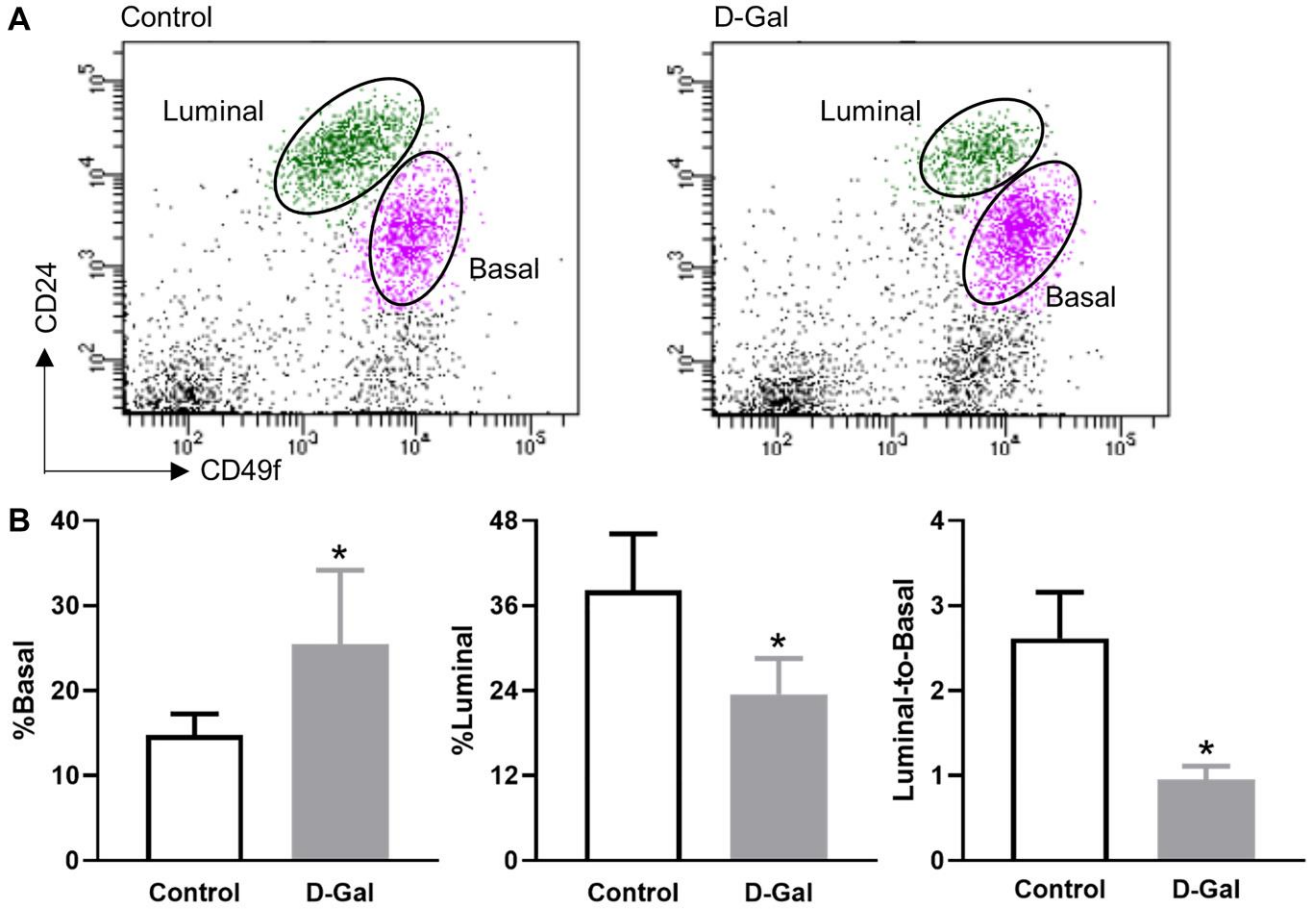
**D-galactose alters mammary basal/luminal cell pools.** (**A**) Representative flow cytometry analysis of mammary epithelial cells from control and D-galactose-treated mice. Basal cells express high levels of CD49f, and luminal cells express high levels of CD24; (**B**) Quantification of % basal cell, % luminal cell, and luminal-to-basal cell ratio in mammary epithelial cells isolated from control and D-galactose-treated mice (*n* = 5). Asterisks, significant difference between control and D-galactose (^*^P < .05).

Mammary epithelial cells are composed of basal and luminal cells. Flow cytometry analysis revealed increased basal cells (CD24^low^CD49^high^) and decreased luminal cells (CD24^high^CD49^low^) in mammary glands harvested from D-galactose-treated mice when compared to control mice (Figure 3A). Quantitative analysis showed that the frequency of basal cells increased from 14.8 ± 2.4% in the control mice to 25.5 ± 8.7% in the D-galactose-treated mice (Figure 3B). In contrast, the frequency of luminal cells decreased from 38.2 ± 7.9% in control mice to 23.5 ± 5.1% in D-galactose-treated mice (Figure 3B). As a consequence, the luminal-to-basal cell ratio decreased from 2.6 ± 0.5 in control mice to 1.0 ± 0.2 in D-galactose-treated mice (Figure 3B).

### D-galactose alters stem/progenitor cell function *in vitro*

**Figure 4:**
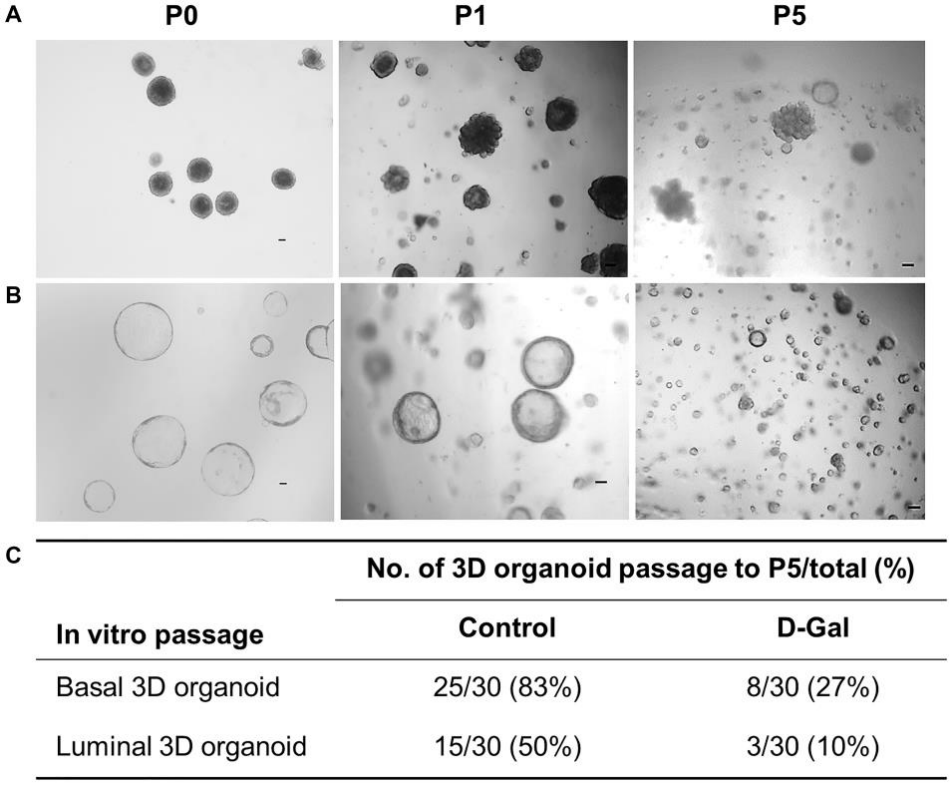
**D-galactose alters mammary stem/progenitor cell function *in vitro*.** (**A**) Representative images showing *in vitro* serial passage of basal stem cell contained 3D organoids from primary organoids (P0) to P1 and P5 (scale bars, 100 μm); (**B**) Representative images showing *in vitro* serial passage of luminal progenitor cell contained 3D organoids from primary organoids (P0) to P1 and P5 (scale bars, 100 μm); (**C**) Quantification of the number of 3D organoids that can be passaged to P5 for stem/progenitor cells derived from control and D-galactose-treated mice (for each type of 3D organoids, 10 organoids per animal × 3 animals = 30 organoids were assayed).

Mammary epithelium is orchestrated by a hierarchy of multipotent and unipotent stem cells; however, it is generally agreed that unipotent basal and luminal stem cells are responsible for routine epithelial maintenance [21, 22]. In this study, we refer to stem cells enriched in the basal cell fraction as mammary stem cells (MaSCs) and those enriched in the luminal cell fraction as luminal progenitors (LPs) to be consistent with existing literature. Both MaSCs and LPs were assessed for their *in vitro* self-renewal function using the 3D organoid serial passage assay. To obtain 3D organoids, sorted basal or luminal cells were first allowed to form spheres in suspension culture and then transferred to 3D Matrigel culture for sphere differentiation. The 3D organoids formed by spheres exhibited distinct morphological differences between MaSCs and LPs, with the former forming solid structures and the latter forming hollow-like structures [23]. These individual 3D organoids were dissociated into a single cell suspension and passaged *in vitro* for five generations (Figure 4A, 4B). For MaSCs, out of 30 basal 3D organoids in the control mice, 25 of them (83%) were able to be passaged to P5, while only 27% of basal 3D organoids from D-galactose-treated mice successfully reached P5 (Figure 4C), indicating declined self-renewal function. Similarly, for LPs, the number of 3D organoids that could be passaged to P5 decreased from 50% in the control mice to 10% in the D-galactose-treated mice (Figure 4C).

**Figure 5:**
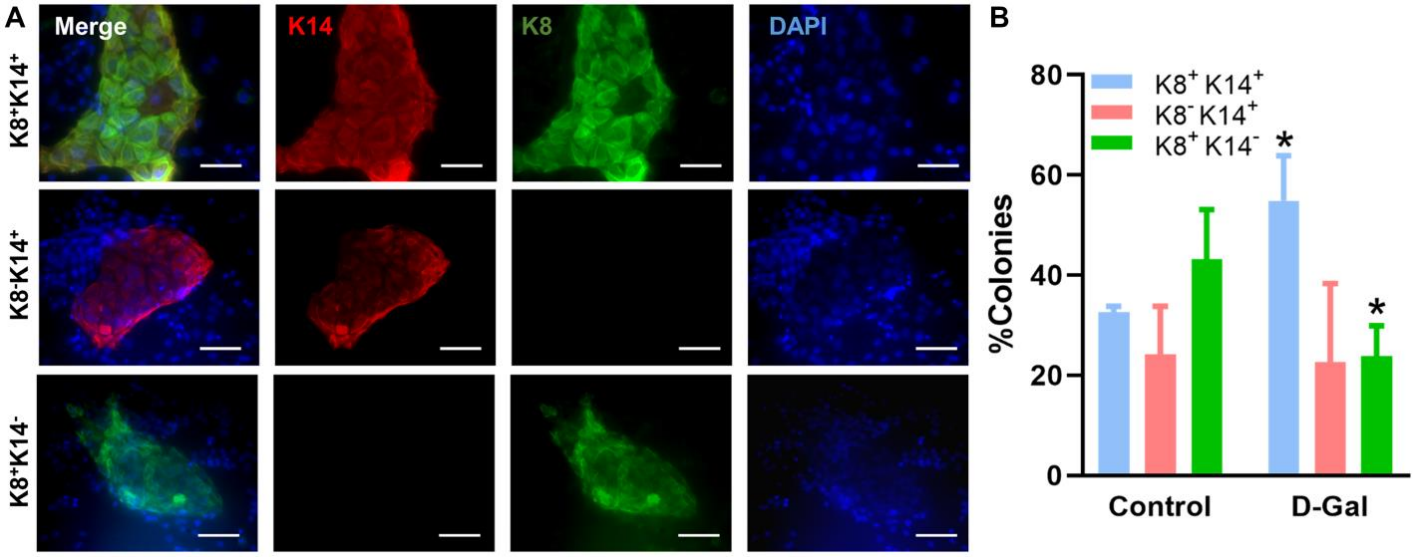
**D-galactose alters luminal progenitor cell differentiation *in vitro*.** (**A**) Representative immunofluorescence images showing distinct colonies formed by luminal progenitor cells on plates pre-seeded with irradiated NIH-3T3 fibroblasts; (**B**) Bar graph shows the distribution of different types of colonies formed by luminal progenitor from control and D-galactose-treated mice (> 20 colonies per animal × 3 animals). K14: basal cell marker keratin 14; K8: luminal cell marker keratin 8.

We also characterized LP differentiation *in vitro* using the 2D colony forming assay, where LPs were found to form morphologically distinct colonies when cultured on plates pre-seeded with irradiated NIH-3T3 fibroblasts (Figure 5A). Colonies formed by LPs from D-galactose-treated mice were dominated by K8^+^K14^+^ bipotent colonies (55 ± 9%), while those from control mice were dominated by K8^+^K14^–^ colonies (43 ± 10%) (Figure 5B). In fact, there was a significant reduction of K8^+^K14^–^ colonies (24 ± 6%) in D-galactose-treated mice. The percentage of colonies displaying K8^–^K14^+^ was similar between control (24 ± 10%) and D-galactose-treated mice (23 ± 16%) (Figure 5B). MaSC differentiation was not assessed *in vitro* because they cannot form colonies in this assay.

### D-galactose alters stem/progenitor cell function *in vivo* and increases hyperplasia in regenerated glands

**Figure 6:**
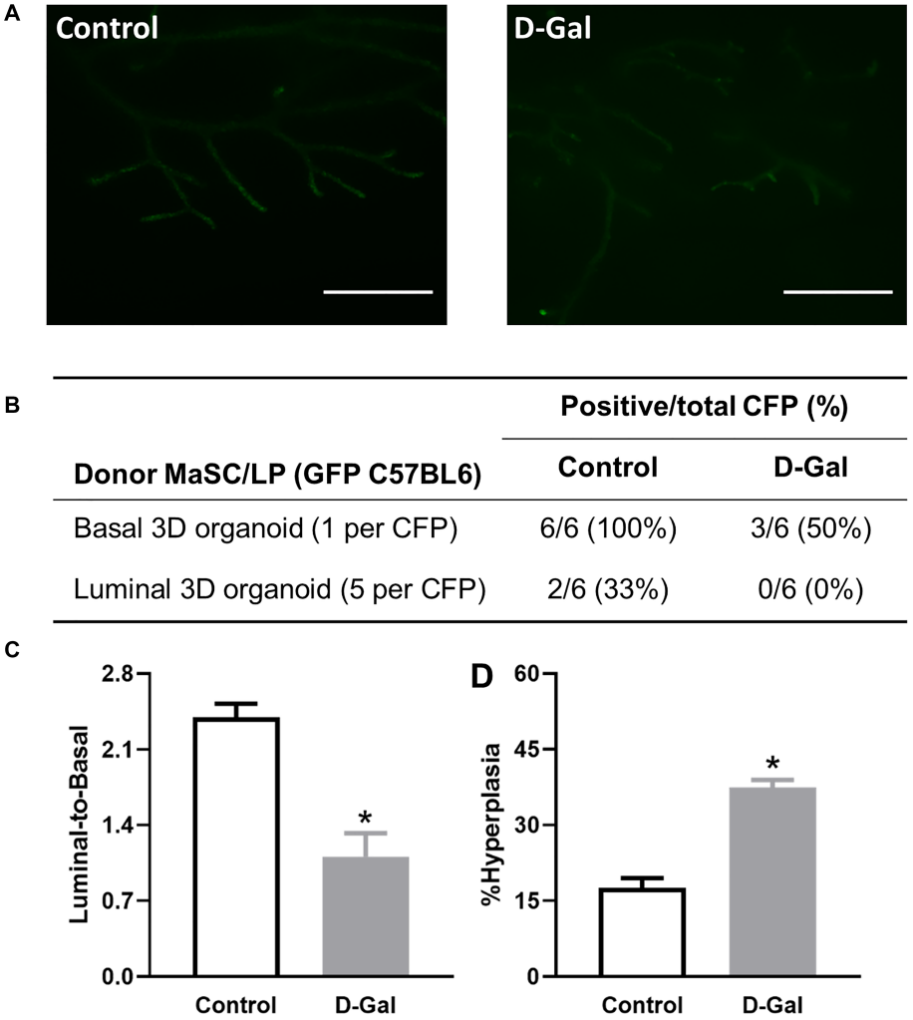
**D-galactose alters mammary stem/progenitor cell function *in vivo* and increases hyperplasia in regenerated glands.** (**A**) Regenerated glands from *in vivo* transplant of one basal 3D organoid from GFP mice with or without D-galactose treatment (scale bars, 1 mm); (**B**) Positive take of outgrowth from control or D-galactose-treated stem/progenitor cells in the cleared fat pad (CFP) transplant assay; (**C**) Luminal-to-basal cell ratio in mammary epithelial cells isolated from regenerated glands derived from control and D-galactose-treated GFP mice (*n* = 3–4); and (**D**) H&E histology analysis shows % hyperplasia in mammary ducts from regenerated glands derived from control and D-galactose-treated GFP mice (*n* = 3–6). Asterisks, significant difference between control and D-galactose (^*^P < .05).

We further assessed stem cell self-renewal and differentiation by the cleared fat pad (CFP) transplant assay. Transgenic GFP C57BL/6 mice were treated with control or D-galactose, and MaSCs and LPs were subsequently isolated as donors for *in vivo* transplant. We have previously shown that 3D organoids contain stem cells that can repopulate CFP [23]. Thus, 3D organoids were used for *in vivo* transplantation. Regenerated glands were clearly visible with the fluorescence microscope given their expression of GFP (Figure 6A). For MaSCs, upon transplant of one single 3D organoid per CFP, we obtained six positive outgrowths from six transplants in the control MaSCs, representing 100% engraftment frequency, but only three positive outgrowths (50% engraftment) in MaSCs derived from the D-galactose-treated mice (Figure 6B). For LPs, we injected five individual 3D organoids per CFP into six CFPs and obtained two positive outgrowths (33% engraftment) in the control LPs, but none in the D-galactose-treated LPs (Figure 6B). The regenerated glands were further harvested for flow cytometry analysis of the luminal-to-basal cell ratio as well as histopathological analysis. Our analysis showed a decreased luminal-to-basal ratio of regenerated glands from D-galactose-treated MaSCs (1.1 ± 0.2) when compared to those from control MaSCs (2.4 ± 0.1) (Figure 6C). Similar to primary mammary glands, regenerated mammary ducts formed by D-galactose-treated MaSCs also showed a higher proportion of hyperplastic ducts than those from control MaSCs (37.5 ± 1.5% vs. 17.6 ± 1.9%) (Figure 6C).

## DISCUSSION

The present study showed that mammary glands in a D-galactose-treated mouse model are characterized by enlarged lymph nodes, increased expression of p-STAT5, decreased branching points, increased hyperplasia, increased basal cell pool comprised of mostly CD49f^high^ cells, reduced luminal-to-basal cell ratio, and decreased stem/progenitor cell self-renewal function. Interestingly, LP *in vitro* differentiation-formed colonies were dominated by K8^+^K14^+^ bipotent colonies in D-galactose-treated mice instead of K8^+^K14^–^ colonies in the control group. Regenerated glands from D-galactose-treated stem cells continuously displayed the altered luminal-to-basal cell ratio and increased hyperplasia phenomena, as in the primary glands. In addition, increased inflammatory cytokine signals were found in serum samples from D-galactose-treated mice. Most of these observations phenocopied the changes we observed in mammary glands from old C57BL/6 mice (25–32 months) in our earlier study [13], revealing that the D-galactose-induced accelerated aging model is similar to natural aging in mammary glands and stem cell function. These findings suggest that the D-galactose-induced aging model can be used to study aging-related breast cancer etiology, initiation, progression, and potential therapeutic interventions.

Mammary glands in old mice from natural aging are characterized by increased tertiary structures, increased hyperplastic lesions, expanded basal cell pool, and altered luminal-to-basal cell ratio [13]. Except for morphological appearance, mammary glands from D-galactose-treated mice essentially recapitulate all other changes found in old mice. For example, D-galactose-induced hyperplasia (43%) is similar to what we observed in old C57BL/6 mice (44%) [13]. Similarly, epithelial cell composition is comparable between D-galactose-treated mice and old C57BL/6 mice in terms of % of basal cells (25.5% vs. 28.4%), % of luminal cells (23.5% vs. 16.8%), and luminal-to-basal ratio (1.0 vs. 1.1) [13]. All these values are very similar between the control mice (aged 4.5 months) in this study and the young C57BL/6 mice (2–4 months) in our previous study [13]. This good concordance is expected given the same mouse strain with a similar age range employed in both studies. By using the same mouse strain, we demonstrated that D-galactose-induced changes in mammary gland pathology and epithelial cell composition are consistent with the changes caused by natural aging.

We further examined mammary stem cell function in the D-galactose-induced aging model. It is known that mammary stem cells display decreased self-renewal with aging [13]. In the present study, we first evaluated MaSC/LP function using *in vitro* assays. Serial passage of cells dissociated from a single 3D organoid revealed decreased self-renewal potential for both MaSCs and LPs after D-galactose treatment. When compared to basal 3D organoids, luminal organoids showed decreased self-renewal potential in both control and D-galactose-treated mice. This finding is consistent with the more limited self-renewal potential associated with LPs found in the cleared fat pad transplant assay of this study and those of others [24, 25]. For LPs, an *in vitro* colony forming assay was used to assess its differentiation function. Interestingly, with D-galactose treatment, colonies formed were mainly dominated by K8^+^K14^+^ bipotent colonies instead of the K8^+^ luminal-restricted colonies observed in the control mice, suggesting altered LP subpopulations. Coincidentally, gene enrichment analysis and immunofluorescence staining in our earlier study indicated a potential mechanism by which luminal cells undergo luminal-to-basal phenotypic changes during aging [13]. It therefore seems that D-galactose and natural aging altered LP differentiation in a similar way. We did not evaluate MaSC differentiation *in vitro*, as the colony forming assay is not applicable to basal stem cells [23].

To test mammary stem cell function *in vivo*, the cleared fat pad transplant assay was generally used [25]. With natural aging, mammary stem cells not only display a functional decline, but also an increased transformation potential [13]. In the D-galactose-induced aging model, both MaSCs and LPs showed decreased engraftment success when compared to their control counterparts. It is noteworthy that the cleared fat pad transplant assay is usually only used to assay MaSC repopulating potential *in vivo* because LPs cannot repopulate the CFP as efficiently as MaSCs [24, 25]; however, our own findings showed significant engraftment success when LPs were injected into the CFP in the form of 5 or more 3D organoids (unpublished data). Nevertheless, LPs consistently displayed lower engraftment than MaSCs in this *in vivo* transplant assay. With the regenerated glands from D-galactose-treated MaSCs, we further assessed their epithelial cell composition and histopathological changes. Interestingly, the regenerated glands from D-galactose-treated MaSCs maintained an altered luminal-to-basal cell ratio, as in D-galactose-treated primary glands. In addition, these regenerated glands also showed a higher frequency of hyperplastic lesions when compared to control MaSC-derived outgrowth, revealing similar changes as we observed in regenerated glands from old MaSCs [13]. Therefore, both *in vitro* and *in vivo* MaSC/LP functional analysis showed similar changes in the D-galactose-induced aging model as the naturally aged mice.

One important hallmark associated with aging is chronic inflammation [26]. In particular, aging and many aging-related diseases are characterized by cytokine dysregulation. For example, IL-6 is considered to be the leading biomarker of aging-related diseases, and over production of IL-6 may contribute to tissue aging and function decline [27–30]. Interestingly, inflammation is also an important hallmark for various organs in D-galactose-induced aging models [17]. In the present study, D-galactose treatment caused a significant increase in pro-inflammatory cytokines of IL-6 and TNFα in serum samples, indicating an elevated systemic inflammatory response. This finding was consistent with earlier studies using D-galactose-induced aging models [31, 32]. In addition, we also observed enlarged lymph nodes and enhanced expression of p-STAT5 in the mammary glands from D-galactose-treated mice, suggesting a tissue-specific inflammatory response in the local environment. It is known that STAT5 can be activated by cytokines such as IL-6 and TNFα, and persistent activation of STAT5 can promote chronic inflammation, which subsequently increases cell transformation potential [33]. In our earlier comparative study between young and old mammary glands, whole transcriptome analysis revealed activated inflammatory signals, immune responses, and elevated p16 expression in old mammary glands, which are hypothesized to be the underlying causes for the altered epithelial cell composition, stem cell frequency, and function observed in old mice [13]. We suspect similar underlying causes are responsible for D-galactose-induced mammary stem/progenitor cell changes, as elevated inflammatory signals, immune responses, and p16 expression were also observed in the D-galactose-induced aging model [17]. Future studies are necessary to confirm this hypothesis.

In conclusion, mammary glands in the D-galactose-induced aging model mimic natural aging in terms of pathological changes, epithelial cell composition, and mammary stem/progenitor cell function. These changes were accompanied by elevated inflammatory responses both systemically in the blood and locally in the mammary glands, which were also similar to naturally aged mice. Our study for the first time evaluated the mammary glands and mammary stem/progenitor function in a D-galactose-induced aging model in rodents, and our findings suggest that D-galactose treatment can be used as a valid tool to mimic natural aging in breast cancer research.

## MATERIALS AND METHODS

### Animals

Animal care and use were conducted according to established guidelines approved by the Institutional Animal Care and Use Committee at Wenzhou Medical University (Approval number: 2018-211). Wild type C57BL/6 mice, purchased from Nanjing Medical University, were housed at 20–25°C in a light-controlled (12/12 h) room with unrestricted access to water and food. Transgenic green fluorescent protein (GFP) C57BL/6 mice were originally obtained from Jackson Laboratory and raised in our facility.

### D-galactose aging model

Accelerated aging in mice was induced by daily subcutaneous injection of 100 mg/kg of D-galactose for 45 days. The control mice were injected with phosphate buffer saline (pH 7.4). The injection was started at three months old and five mice were used for each treatment group. For the *in vivo* transplant experiment, GFP C57BL/6 mice were used for control or D-galactose treatment.

### Enzyme-linked immunosorbent assay (ELISA)

Blood was collected by venipuncture and allowed to clot at room temperature. Serum was collected after centrifugation at 700× g for 10 min and stored at −80°C before use. The levels of IL-6 and TNF-α were measured using corresponding ELISA kits from Abcam according to user instructions, and the OD value was read at 450 nm using a BioTek spectrophotometer.

### Quantification of ductal hyperplasia

Normal ductal structures are characterized by an outside myoepithelial cell layer and an inside luminal epithelial cell layer. Hyperplastic ducts are characterized by more than two layers of epithelial cells (Figure 2B). The percentage of hyperplasia was calculated by dividing the number of ductal structures showing hyperplastic lesions by the total number of ducts counted. A minimum of 50 ductal structures were counted for each gland.

### Immunohistochemistry

Mammary glands were fixed for 36 h in 10% neutral-buffered formalin, dehydrated in ethanol, and embedded in paraffin wax. Tissue sections of 4 μm on glass slides were processed as described previously [13]. After antigen retrieval and blocking of nonspecific binding, sections were incubated with recombinant anti-STAT5 (phosphor Y694) antibody [E208] (ab32364, Abcam) (dilution 1:100) overnight at 4°C, and then washed and incubated with biotin conjugated secondary antibodies for 1 h at room temperature. Sections were further incubated with streptavidin-horseradish peroxidase for 30 min and stained with diaminobenzidine for 15 min before dehydration and mounting.

### Whole-mount carmine staining

Mammary glands were dissected, spread onto glass slides, and fixed overnight in Carnoy’s fixative at room temperature and processed as previously described [13].

### Mammary epithelial cell preparation and flow cytometry analysis

Mammary inguinal and thoracic glands were harvested and processed to generate single cell suspensions for flow cytometry (FACSAria-IIIu, BD Biosciences) analysis as previously described [23, 34]. Mammary stem/progenitor cells were enriched and isolated from endothelial (CD31) and hematopoietic (CD45 and TER119) lineage-depleted (Lin^-^) mammary epithelial cells using cell surface markers CD24 and CD49f. Basal stem cells are enriched in basal cells characterized by CD24^low^CD49f^high^ and luminal stem cells are enriched in luminal cells characterized by CD24^high^CD49f^low^.

### *In vitro* serial passage of 3D organoids

Sorted basal and luminal cells were first used for mammary sphere culture and subsequently followed by sphere differentiation in Matrigel 3D culture [23]. To assess stem cell self-renewal function *in vitro*, 3D organoids formed in Matrigel culture (P0) were used for *in vitro* serial passage as we described previously [35]. The serial passage process was repeated for five generations (P5), and the number of colonies that can be continuously passaged to P5 was tabulated against the total number of colonies assessed. For each type of 3D organoids (basal or luminal), 10 organoids per animal × 3 animals, a total of 30 organoids were assayed.

### Mammary colony forming cell assay

The mammary colony forming cell assay is used to assess the differentiation function of luminal progenitor cells. It is known that luminal progenitor cells can form distinct colonies when cultured on plates pre-seeded with irradiated NIT-3T3 fibroblasts [23, 35]. In this study, the colony forming cell assay was performed [13]. However, instead of Giemsa staining, colonies were stained with basal and luminal markers of keratin 14 (K14) and K8, respectively, using immunofluorescence (IF). In detail, colonies were fixed with 100% cold methanol for 1 min, washed with phosphate buffer solution with 0.05% Tween 20 (PBST), blocked with 10% serum/1%BSA/0.3M glycine in 0.1% PBS-Tween 20 for 30 min, and incubated with primary antibodies of K14 (rabbit monoclonal IgG, ab119695, 1:200 dilution, Abcam) and K8 (rabbit monoclonal IgG, ab53280, 1:200 dilution, Abcam) diluted in 5% serum/1%BSA/0.3M glycine in 0.1% PBS-Tween 20 at 4°C overnight. Colonies were then washed with PBST twice before being incubated with secondary antibodies diluted in 1%BSA/0.3M glycine in 0.1% PBS-Tween 20 at room temperature for 1 h. Finally, colonies were washed with PBST and stained with DAPI for fluorescent imaging. A minimum of 20 colonies per animal (× 3 animals) per treatment group were assessed with IF.

### Cleared fat pad transplant

Stem cell self-renewal and differentiation *in vivo* was assessed by the cleared fat pad transplant assay as described previously [13]. In brief, 3D organoids from control and D-galactose-treated GFP C57BL/6 mice were injected into the contralateral inguinal glands of 21-day-old virgin wild-type female C57BL/6 mice cleared of endogenous epithelium. For basal stem cells, one single solid 3D organoid was injected into one cleared fat pad (CFP). For luminal progenitor cells, five hollow 3D organoids were injected into one CFP. A total of six CFPs were used for each type of 3D organoid per treatment group. Outgrowths, defined as epithelial structures with both ductal and lobular structures, were evaluated by Nikon fluorescence microscope after eight to ten weeks. The regenerated glands were further harvested for flow cytometry analysis of the luminal-to-basal cell ratio as well as histopathological analysis for hyperplastic lesions.

### Statistical analysis

Data were analyzed using GraphPad Prism 6.0 (GraphPad Software Inc., La Jolla, CA). Differences between various treatment groups were evaluated with a non-paired *t*-test. Results are presented as mean ± SD. Statistical significance was defined by *P* < .05.
